# Unmet medical needs in ambulatory care in Hungary: forgone visits and medications from a representative population survey

**DOI:** 10.1007/s10198-019-01063-0

**Published:** 2019-05-17

**Authors:** Armin Lucevic, Márta Péntek, Dionne Kringos, Niek Klazinga, László Gulácsi, Óscar Brito Fernandes, Imre Boncz, Petra Baji

**Affiliations:** 1 Department of Health Economics, Corvinus University of Budapest, Fővám tér 8, Budapest, 1093 Hungary; 2 Department of Public Health, Amsterdam UMC, University of Amsterdam, Amsterdam Public Health Research Institute, Meibergdreef 9, 1105 AZ Amsterdam, The Netherlands; 3 Institute for Health Insurance, Faculty of Health Sciences, University of Pécs, Mária u. 5-7, Pécs, 7621 Hungary

**Keywords:** Forgone care, Unmet medical needs, Access, Ambulatory care, Hungary, I11, I13, I14

## Abstract

**Background:**

The objective of this paper is to explore unmet health care needs in Hungary in ambulatory care due to costs and difficulties in travelling, and to analyze how unmet needs relate to socio-demographic characteristics.

**Methods:**

The quantitative analysis is based on a national, representative online survey carried out in Hungary on a sample of 1000 respondents in early 2019 using a proposed set of questions developed by the OECD. We present and compare unmet medical needs in different socio-demographic groups, and we use multivariate logistic regression analysis to identify the main determinants of unmet medical needs.

**Results:**

Among responders who had medical problems in the last 12 months, 27.3% reported forgone medical visit due to difficulties in travelling, 24.2% had unfilled prescription for medicine due to costs, 21.4% reported forgone medical visit or follow-up visit due to costs and 16.6% reported skipped medical test, treatment or other follow-up due to costs. These shares are much higher than presented previously in international databases. The logistic model indicates that respondents were significantly more likely to report unmet needs if they were women, younger or belonged to first and second income quintiles.

**Conclusions:**

Policy makers need to address the issue of high prevalence of forgone medical care among the Hungarian population to avoid deterioration of population health and inequalities in access. As a first step, policies should try to decrease financial burden of vulnerable groups to improve access.

## Introduction

In the Health System Performance Assessment (HSPA) framework, developed by World Health Organization (WHO) in 2007, access to health care is a key performance domain for health care system performance, and a critical component of universal health coverage [1]. Equitable access to (good quality) care is also recognized to be one of the common values of the European Union (EU) [2], as access to effective health care by those in need improves health [2], prolongs life and prevents suffering; and improved population health drives economic growth, greater labor force participation and higher productivity [3].

Unmet medical needs (or forgone care used here as a synonym),^1^ as an indicator of the lack of access, present a situation in which patients are unable to obtain adequate access to health services they need, due to cost, distance or waiting lists [4]. Unmet medical needs may not just result in poorer health status of the population, but contribute to increasing health inequalities as well, as most often, vulnerable social groups have the most difficulties with access [5]. Therefore, the EU has been committed to monitor unmet medical needs [data are collected in two Eurostat surveys, the European Union Statistics on Income and Living Conditions (EU-SILC) [6] and the European Health Interview Survey (EHIS) [7]] and to stimulate to reduce their level.

In Hungary, the healthcare system is based on universal health coverage, where most of the health care services are provided free of charge [8], however, rising out-of-pocket payments exceeds 30% of the total health expenditure [9]. In 2014, 22.5% of the Hungarian population reported unmet needs for health care, 13.8% due to financial reasons, 12.8% due to waiting lists and 2.6% due to distance or transportation [10]. This percentage is below the EU average (26.5%), but higher than in some other Central-Eastern European (CEE) countries such as the Czech Republic (17.3%), Slovakia (11.4%), or Romania (15.5%) [10]. Among CEE countries, people reported the most unmet medical needs in Slovenia (26.1%) and Poland (32.3%) [10]. Furthermore, according to the European Quality of Life Surveys (EQLS) data from 2016 for Hungary, 47% of the responders have difficulties (very difficult 11%) to see the doctor due to waiting time, 18% due to distance to the doctor’s office and 15% of the responders are having difficulties to see the doctor due to financial reason. In general, unmet needs for medical care have decreased since 2015 on average across EU countries [9] but burden still falls mostly on people with low-income (in the lowest income quintile) and the elderly population (age 65 and more) [11].

Among OECD countries, Hungary is still lacking behind to collect patient experience data on system level and use international benchmarking to enhance healthcare improvements and reforms [12]. So far, an in-depth analysis on the determinants of unmet medical needs in ambulatory care is missing for Hungary. Also, there are certain limitations when comparing the data among different subgroups of the population in existing datasets, as most comparisons for accessibility are based on age, gender; and in some cases on income, education and urbanization level, but no data are available by different health status for example.

The objective of this paper is to study unmet health care needs of the Hungarian population focusing on access to ambulatory care, specifically on forgone visits and medication due to costs and difficulties in travelling, and to analyze how unmet needs relate to patient/person characteristics. In our study, we will also pay attention to international comparisons, especially with other OECD countries and CEE countries.

The quantitative analysis is based on a national, representative online survey carried out in Hungary on a sample of 1000 respondents in early 2019. The results of our study are expected to raise the importance of healthcare system performance measurement and patient empowerment in Hungary, furthermore, might help policy makers to monitor and improve access to ambulatory care and decrease inequalities in the utilization of health care services.

## Methods

### Study design and population

The online (web administered) survey on “Patient experiences in healthcare”, was performed in early 2019. The data collection was carried out by Big Data Scientist Kft. Respondents were selected from a large online panel of the survey company. Quotas were applied based on the last census in Hungary in 2011 to achieve a sample of 1000 respondents, representative for the Hungarian adult population in terms of gender, age (by age-groups of 18–24, 25–34, 35–44, 45–54, 55–64 and a reasonable sample for age 65 and over), educational level (primary, secondary, tertiary), type of settlement (capital, town, village) and region (Central, Eastern, Western Hungary).

Ethical approval was obtained by the Medical Research Council of Hungary (47654–2/2018/EKU). Before completing the questionnaire, respondents gave their informed consent and were informed about the objective of the survey, about their voluntary participation, and that the data collected would be used anonymously and for research purposes only.

### Survey and definition of variables

To measure patient experience with ambulatory care we used a proposed set of questions developed by the OECD in 2010. These questions are based on the International Health Policy Survey conducted on a regular basis by the Commonwealth Fund in 11 countries [12]. To develop the Hungarian version of the questions, forward–backward translation and cognitive testing were applied. The questionnaire was piloted with five respondents, involving an interviewer. Respondents were asked to complete the pilot questionnaire in the presence of the interviewer to be able to raise questions or ask for explanations if it was necessary. After that, the interviewer and respondent discussed the statements.

In this study we focus on questions related to the unmet medical needs of patients during the last 12 months, specifically: if patients (1) reported forgone medical visit or follow-up appointment due to costs; (2) forgone medical test, treatment or other follow-up due to costs; (3) unfilled prescription for medicine, or skipped doses due to costs; (4) forgone medical visit due to difficulties in travelling.

### Analysis

For statistical analyses, we used software Stata 14. No identifiable information was used when the data were processed. We present and compare unmet medical needs in socio-demographic groups by age (18–24; 25–34; 35–44; 45–54; 55–64; 65 + ), gender (men/women), marital status (married or partnership/not married), employment status (working full/part time/no paid job), self-perceived health (excellent; very good; good; fair; poor), region (central; east; west), type of residence (Budapest; town; village), education level (primary or less; secondary; tertiary) and household income (five quintiles). Income quintile groups were created based on the net total equivalized disposable income attributed to each member of the household. The first quartile represents 20% of the population with the lowest income, and the fifth quintile represents 20% with the highest income.

To better represent the Hungarian population, sampling weights were applied for the calculations considering gender, age, education level, type of residence, and region. In all statistical analyses, weights were applied. Due to this fact, instead of Chi-square statistic, we used design-based *F*-statistic which gives more accurate *p* values for weighted samples. We used multivariate logistic regression analysis to identify the main determinants of unmet medical needs. 95% confidence intervals (robust) were calculated.

In our analyses, we excluded those who (1) did not report medical problems in the last 12 months (11.0% for all questions) (2) declined to answer the questions (2.3%, 2.2%, 1.8%, and 2.2% for the four questions, respectively), or (3) did not remember (1.9%, 1.9%, 1.4%, and 2.2%).

## Results

Out of the 1000 respondents, 89.0% reported medical problems in the last 12 months. Among them (excluding those who declined to answer or did not remember), 21.4% reported forgone medical visit or follow-up visit due to cost, 16.6% reported skipped medical test, treatment or other follow-up due to costs, 24.2% unfilled prescription for medicine, or skipped doses due to costs, and 27.3% forgone medical visit due to difficulties in travelling (Table 1).

**Table 1:** Descriptive statistics and unmet medical needs by subgroups

Variables	Total sample (*N* = 1000)	Share of those who reported forgone medical visit to follow-up appointment due to costs (*N* = 848)		Skipped medical test, treatment or other follow-up due to costs (*N* = 849)		Unfilled prescription for medicine, or skipped doses due to costs (*N* = 859)		Forgone medical visit due to difficulties in travelling (*N* = 846)	
Total	100%	21%		16.6%		24.2%		27.3%	
Age category									
18–24	10.6%	25.7%	***F*** **= 3.6587** ***p*** **= 0.0029**	12.1%	*F* = 0.6915 *p* = 0.6265	32.8%	***F*** **= 1.8995** ***p*** **= 0.0928**	33.6%	*F* = 1.5092 *p* = 0.1847
25–34	16.9%	31.8%		19.1%		28.8%		32.7%	
35–44	18.8%	25.8%		20.4%		19.4%		27.5%	
45–54	15.5%	17.5%		14.5%		29.1%		28.0%	
55–64	17.6%	17.3%		16.2%		23.3%		28.1%	
65 +	20.6%	13.0%		15.3%		18.9%		19.5%	
Gender									
Women	53.4%	26.4%	***F*** **= 13.9595** ***p*** **= 0.0002**	18.7%	*F* = 2.6076 *p* = 0.1067	29.8%	***F*** **= 13.707** ***p*** **= 0.0002**	32.4%	***F*** **= 10.8383** ***p*** **= 0.0010**
Men	46.6%	14.8%		14.2%		17.7%		21.1%	
Marital status									
Married/partner	62.9%	20.7%	*F = 0.0478* *p = 0.8269*	15.8%	*F* = 0.7174 *p* = 0.4430	23.3%	*F* = 0.5765 *p* = 0.4479	24.7%	*P* = 0.0438 *F* = 4.0770
Not married	37.1%	21.5%		18.1%		25.9%		31.8%	
Employment status									
Working full/part time	48.5%	20.9%	*F* = 0.0088 *p* = 0.9255	15.3%	*F* = 0.8936 *p* = 0.3448	21.8%	*F* = 2.1720 *p* = 0.1409	25.5%	*F* = 1.0242 *p* = 0.3118
No paid job	51.5%	21.2%		17.9%		26.6%		28.9%	
Health status (self-perceived health)									
Excellent	7.38%	22.9%	***F*** **= 2.8717** ***p*** **= 0.0224**	12.0%	***F*** **= 2.9610** ***p*** **= 0.0190**	23.2%	***F*** **= 5.2587** ***p*** **= 0.0004**	16.4%	***F*** **= 7.0155** ***p*** **= 0.0000**
Very good	26.9%	18.6%		13.0%		15.1%		20.9%	
Good	38.4%	17.2%		14.0%		2.0%		23.3%	
Fair	23%	26.1%		22.8%		35.7%		36.0%	
Poor	4.4%	37.7%		29.0%		30.4%		55.5%	
Regions									
Central Hungary	30%	22.3%	*F* = 0.1628 *p* = 0.8486	15.5%	*F* = 0.8761 *p* = 0.4162	23.0%	*F* = 0.3064 *p* = 0.7348	29.3%	*F* = 0.5378 *p* = 0.5829
Eastern Hungary	39.6%	20.8%		18.8%		25.7%		27.5%	
Western Hungary	30.4%	20.1%		14.8%		23.4%		24.8%	
Type of residence									
Budapest	18.1%	21.3%	*F* = 1.2199 *p* = 0.2951	12.1%	*F* = 1.1963 *p* = 0.3021	20.3%	*F* = 1.4093 *p* = 0.2447	26.7%	*F* = 1.9152 *p* = 0.1489
Town	51.9%	19.0%		17.3%		23.5%		24.7%	
Village	30%	24.7%		18.3%		28.0%		32.2%	
Education level									
Primary or less	51%	23.4%	***F*** **= 4.5277** ***p*** **= 0.0126**	19.0%	***F*** **= 2.7905** ***p*** **= 0.0651**	28.4%	***F*** **= 10.215** ***p = 0.0001***	32.5%	***F*** **= 10.2681** ***p*** **= 0.0001**
Secondary	31.3%	22.0%		15.9%		24.7%		26.0%	
Tertiary	17.7%	12.5%		11.1%		11%		14.5%	
Household income category									
1st quintile	28.2%	30.4%	***F*** **= 4.9082** ***p*** **= 0.0007**	22.6%	***F*** **= 3.4408** ***p*** **= 0.0085**	31.3%	***F*** **= 4.0492** ***p*** **= 0.0030**	35.7%	***F*** **= 4.3033** ***p*** **= 0.0019**
2nd quintile	18.8%	25.7%		23.1%		32.7%		36.6%	
3rd quintile	20.0%	15.0%		11.0%		24.1%		21.1%	
4th quintile	19.1%	21.9%		17.6%		21.8%		23.4%	
5th quintile	13.9%	9.9%		8.7%		11.5%		18.2%	
**Total**									

Sample weights were applied for calculations. Significant differences (*p* < 0.1) are highlighted with bold. Primary level of education included those who had fully completed primary education and who partly completed secondary education without direct access to post-secondary or tertiary education. In our analyses, we did not include those who (1) did not report medical problems in the last 12 months (11.0% for all questions) (2) declined to answer the questions (2.3%, 2.2%, 1.8%, and 2.2% for the four questions, respectively), or (3) did not remember (1.9%, 1.9%, 1.4%, and 2.2%)

The highest share of users who reported forgone medical visit or skipped follow-up appointment due to costs is seen among people between the age of 25–34 (31.8%), with poor health status (37.7%) and in the lowest income quintile (30.4%). Participants between the age of 35–44 (20.42%), with poor health status (28.98%) and in the second income quintile (23.09%) reported the highest number of skipped medical test, treatment or other follow-up due to costs. The highest occurrence of unfilled prescription or skipped doses due to costs is observed in the youngest age group, among the 18–24 years old (32.8%), among participants with poor or fair health status (30.4% and 35.6%) and among those from the first and second income quintiles (31.3% and 32.7%). The highest percentage of users who reported forgone medical visit due to difficulties in travelling is seen in the youngest (18–24) age category (33.57%), with fair health status (36.02%) and among the first and second income quintiles (35.7% and 36.58%).

The results of the multivariate logistic model (Table 2) indicate that respondents are significantly more likely to report forgone medical visit or follow-up appointment due to costs if they are women (OR 1.67), younger (OR 0.98), in poor health status (OR 2.79) or belonging to the first, second and fourth income quintiles (ORs 2.78, 2.58, 2.54, respectively). Forgone medical test, treatment or other follow-up due to costs are significantly more likely among users who are living in towns other than the capital (OR 2.26) and belonging to the first, second or fourth income quintiles (OR 2.29, 2.51 and 2.19, respectively). Participants are significantly more likely to report unfilled prescription for medicine or skipped doses due to costs if they are women (OR 1.76), with primary (or less) (OR 2.71) or secondary (OR 2.60) education, or from the second income quintile (OR 2.45). There is also significant association with age (OR 0.98). Our combined analysis shows that respondents who are younger (OR 0.97), living in towns outside of Budapest are significantly more exposed to forgone visits/medication due to financial reasons (OR 0.27) (due to any of the previous three causes).

**Table 2:** The determinants of unmet medical needs—results of the logistic regression analysis

Outcome variables	Forgone medical visit or follow-up appointment due to costs	Skipped medical test, treatment or other follow-up due to costs	Unfilled prescription for medicine, or skipped doses due to costs	Forgone visit/medication due to costs (any of the previous three)	Forgone medical visit due to difficulties in travelling
Explanatory variables	OR (95% CI)	OR (95% CI)	OR (95% CI)	OR (95% CI)	OR (95% CI)
Age	0.975*** (0.960–0.990)	0.995 (0.979–1.011)	0.983** (0.969–0.997)	0.966*** (0.941–0.991)	0.983** (0.969–0.996)
Gender					
Women	1.671** (1.057–2.642)	1.283 (0.803–2.048)	1.761*** (1.155–2.686)	1.639 (0.872–3.080)	1.627** (1.079–2.453)
Men (reference)					
Marital status					
Not married	1.026 (0.666–1.581)	1.366 (0.868–2.148)	1.107 (0.740–1.654)	0.685 (0.372–1.261)	1.619** (1.097–2.390)
Married (reference)					
Employment status					
Not having a paid job	0.999 (0.629–1.585)	0.970 (0.594–1.585)	1.115 (0.723–1.719)	1.024 (0.537–1.952)	1.097 (0.714–1.685)
Working full/part time (reference)					
Health status					
Excellent (reference)					
Very good	0.851 (0.330–2.193)	1.099 (0.356–3.394)	0.582 (0.222–1.524)	0.813 (0.293–2.255)	1.312 (0.476–3.617)
Good	0.750 (0.294 – 1.911)	0.999 (0.329–3.032)	1.034 (0.417–2.567)	0.504 (0.179–1.420)	1.514 (0.563–4.068)
Fair	1.388 (0.515–3.740)	1.635 (0.522–5.128)	1.993 (0.758–5.240)	0.953 (0.305–2.976)	2.840** (1.014–7.956)
Poor	2.794* (0.822–9.500)	2.489 (0.608–10.18)	1.536 (0.449–5.246)	2.899 (0.695–12.09)	6.552*** (1.845–23.26)
Regions					
Central Hungary (reference)					
Eastern Hungary	0.613 (0.315–1.195)	0.668 (0.341–1.312)	0.761 (0.409–1.416)	0.931 (0.332–2.616)	0.589* (0.319–1.084)
Western Hungary	0.574 (0.283–1.164)	0.487* (0.236–1.007)	0.755 (0.397–1.434)	1.149 (0.396–3.337)	0.542* (0.282–1.040)
Type of residence					
Budapest (reference)					
Town	0.939 (0.455–1.938)	2.257** (1.020–4.991)	1.15 (0.563–2.349)	0.270** (0.0799–0.909)	1.267 (0.636–2.523)
Village	1.117 (0.507–2.459)	1.899 (0.753–4.791)	1.241 (0.572–2.689)	0.390 (0.115–1.324)	1.469 (0.702–3.072)
Education level					
Primary or less	1.588 (0.882–2.860)	1.445 (0.749–2.789)	2.709*** (1.474–4.978)	1.091 (0.500–2.378)	1.876** (1.086–3.242)
Secondary	1.585 (0.894–2.811)	1.438 (0.782–2.642)	2.594*** (1.443–4.664)	1.111 (0.541–2.283)	1.646* (0.971–2.789)
Tertiary (reference)					
Household income category					
1st quintile	2.775*** (1.284–5.998)	2.290* (0.920–5.698)	1.827 (0.874–3.817)	2.666 (0.804–8.837)	1.464 (0.712–3.011)
2nd quintile	2.579** (1.149–5.791)	2.507** (1.010–6.227)	2.450** (1.164–5.159)	2.017 (0.619–6.577)	1.816 (0.871–3.786)
3rd quintile	1.387 (0.634–3.038)	1.116 (0.441–2.823)	1.818 (0.867–3.814)	1.606 (0.554–4.652)	0.953 (0.460–1.976)
4th quintile	2.544** (1.186–5.458)	2.193* (0.913–5.266)	1.778 (0.842–3.757)	2.607* (0.862–7.881)	1.288 (0.637–2.605)
5th quintile (reference)					
Constant	0.295* (0.0846–1.029)	0.0506*** (0.0111–0.231)	0.107*** (0.0321–0.356)	0.363 (0.0641–2.058)	0.150*** (0.0428–0.523)
**Observations**	702	702	710	687	699
**Wald Chi** ^**2**^	64.01	30.55	52.36	136.66	56.85
**Pseudo R** ^**2**^	0.0895	0.0541	0.0855	0.4556	0.0906

Sample weights were applied. Robust 95% confidence intervals (CI) in parentheses; OR odds ratio; *p < 0.1; **p < 0.05; ***p < 0.01. Primary level of education included those who had fully completed primary education and who partly completed secondary education without direct access to post-secondary or tertiary education. In our analyses, we did not include those who (1) did not report medical problems in the last 12 months (11.0% for all questions) (2) declined to answer the questions (2.3%, 2.2%, 1.8%, and 2.2% for the four questions, respectively), or (3) did not remember (1.9%, 1.9%, 1.4%, and 2.2%) or (4) did not provide answer to the income question

In addition, participants who are younger (OR 0.98), not married (or in a relationship) (OR 1.62), with primary (or less) (OR 1.88) or secondary education (OR 1.65) are significantly more likely to skip medical visits due to difficulties in travelling. For individuals with a fair or poor health status, the odds of skipped medical visit due to difficulties in travelling are, respectively, 2.8 and 6.6 times higher than the odds for individuals in excellent health status. Also, people from Eastern or Western Hungary were significantly less likely to report forgone care due to difficulties in travelling (ORs 0.59 and 0.54).

## Discussion

The aim of this study was to analyze unmet healthcare needs of the Hungarian population, i.e., forgone visits and medication in ambulatory care due to costs and difficulties in travelling. The survey was based on a standard set of questions proposed by the OECD. This enables compatibility of our results for international comparisons and benchmarking.

Our results confirm that unmet medical needs are common among the Hungarian population, with 21.4% of participants who had medical problems in the last 12 months reporting forgone medical visits or follow-up visits due to cost, 16.6% reporting skipped medical test, treatment or other follow-up due to costs and 24.2% reporting unfilled prescription for medicine, or skipped doses due to costs. For comparative reasons, we also provide these percentages for the whole population (including also those who did not report medical problems in the last 12 months): 18.6%, 14.7% and 21.5% respectively. Reflecting on the data across OECD from 2016 (using the same questionnaire as us, but no data for Hungary are available for comparison), on average just over one in ten people reported having skipped a consultation due to cost [with the highest percentages recorded in Poland (33%), United States (22.3%) and Switzerland (20.9%)]; and 7.1% of people reported having skipped prescribed medicines due to cost [with the highest shares in the United States (18%) and Switzerland (11.6%)] [13].

Comparing to other data sources on Hungary, our shares are higher than those reported by Eurostat based on EHIS and EU-SILC data (both using different questionnaires than ours). EHIS suggests 13.8% for unmet needs for health care due to financial reason in 2014 [10], while based on EU-SILC survey, unmet needs for medical examination due to high expenses show a decreasing trend in Hungary, 2.2%, 0.9%, 0.6%, 0.3% (from 2015 to 2018, respectively) [6]. Our results are closer to the findings of Tambor et al. (2013) [14] that reports that 29.7% of Hungarian responders forewent outpatient physician services due to inability to pay. Besides the use of different questionnaires, explanation for differences in the results between the surveys can be also explained by the fact that most of the surveys (e.g., the EU-SILC) present percentages which are based on the whole population, including those who did not report medical problems during the last 12 months.

High prevalence of unmet medical needs due to costs in Hungary can be partially explained by the high share of out-of-pocket payments in health care financing. Direct payments, cost-sharing for services outside the benefit package, as well as informal payments, account for 29% of all health spending (EU average at 15%) [15]. The majority of these payments are spent on pharmaceuticals [15], which represent the major share of the total household expenditure on health care (75–85%) [16]. Out-of-pocket payments show an increasing trend in Hungary since the beginning of 2007, when health care reforms were implemented with the main goal to increase co-payments for pharmaceuticals and to introduce co-payments for health care services [16] (although these fees were abolished a year later). In theory, most of the health care services are provided free of charge, however, many people still pay informally for visits. Informal payments estimated to make up at least 2.1% of the total health expenditure in 2015 [15]. Another reason of high out-of-pocket expenses can be the increasing utilization of the private sector among the population, especially among the residents of Budapest. Data collected by Szinapszis Piackutató Ltd. indicates that in 2016, 60% of people living in Budapest used private health care services in comparison to 49% in 2014 [17].

The share of unmet medical needs due to difficulties in traveling is 27.3%, and it is the highest among forgone medical care. In contrast to the EHIS data where this share was 2.6% for the year 2014 [10], and SILC data where the share of unmet needs for medical examination due to distance was stable for the last 3 years, 0.2% (2016–2018) [6]. The Hungarian government tried to address the problem of geographical access to outpatient care between 2010 and 2012, when new outpatient units were built in 20 rural micro-regions [18]. After the project implementation, about 430,000 people were able to access health premises within 20-min by car. On the other hand, according to the estimations, at least for 1.6 million people (16% of the population of Hungary) it still takes more than 20-min by car to get to an outpatient clinic [18].

We identified significant differences in unmet medical needs across different socioeconomic groups. Some of our findings—i.e., that the most vulnerable groups at risk are women, those with primary or secondary education, people with poor health status, those living outside the capital, and from the lowest income quintiles—are in line with previous literature [19] [14] [10]. Nevertheless, we also point out some differences with previous evidence. First, EU-SILC data from Hungary 2014 [10] suggest that people between the ages 55–64 were the most exposed to unmet needs. However, we found that in Hungary, younger population groups (between age 18–44) are more at risk. Second, previous research suggests that unmet medical needs are likely to depend on the level of regional differences in public resources devoted to health, in attitudes and health system governance and number and distribution of health facilities per 10,000 population [14]. Nevertheless, we found no significant differences between the regions of Hungary in terms of forgone medical care due to costs. Still, higher prevalence of forgone visits due to difficulties in travelling in Central Hungary needs further research to explore.

We have to acknowledge some limitations of our study. First, the survey was online-based (web administered), which means that non-internet users (especially older populations or respondents of lower socioeconomic status) did not have a chance to participate. Also, those who were invited but refused to participate in the survey could have different answers to our questions. We believe that non-response to the questions did not affect our results too much, as the share of non-responders (declined to answer, did not remember) were below 4% for each question. It is also important to point out recall bias, as participants were asked about past experiences that happened in the last 12 months. Furthermore, the questionnaire was based on closed questions, responders did not have a chance for additional explanations. Lastly, we examined unmet needs due to cost and difficulties in traveling, but the relationship between the costs and traveling difficulties, and the unmet needs due to waiting list/time were not addressed.

In summary, in our explanatory study, we pointed out that forgoing medical care is a common phenomenon in Hungary. The situation seems more alarming than presented previously in international EU-SILC and EHIS studies. Unmet care needs may result in poorer health for people forgoing care, and potentially increased use of health care services at a later point in time (more costly) [20]. Forgone care may also increase health inequalities, leading to further deterioration of financial and social status of vulnerable groups. Thus, public investment in health care systems is of crucial importance to enhance accessibility of health services, therefore, reduce unmet medical needs of the local population. As it is pointed out by representatives of international organizations such as the OECD and the European Commissioner for Health and Food Safety, policies must prioritize financial protection for groups at risk with regard to access to health care. Protecting the most vulnerable groups should be among the top priorities of policy makers. They are not only exposed to high co-payments (especially for pharmaceuticals), but also informal payments which lead to catastrophic expenditure on their household budget. New programs and policies should go in the direction of the reduction of financial burden of the lowest income groups or implementation of refunding mechanism, where part of the payment would be returned through, for example fiscal measures. Strategies to improve access to care for disadvantaged populations (especially people in poor health status) need tackling non-financial barriers as well [13]. Programs which would enhance better distribution of doctors and health facilities could significantly reduce unmet needs due to traveling. Mobile health clinics proved to be effective in providing better access to health care, particularly for minority groups and aging population living in rural areas [21]. In addition, policy responses should be in line with adequate supply and distribution of the health workforce to the cities other than Budapest, which might be challenging due to the increasing lack of human resources and the emigration of health professionals.

Further research is needed to identify whether forgone medical care is caused by behavioral factors (e.g., higher population expectations) or real barriers, particularly among the younger population.

## Footnotes

1 The Eurostat defined unmet need as follows: “Self-reported unmet needs for medical care concern a person’s own assessment of whether he or she needed examination or treatment for a specific type of health care, but did not have it or did not seek it because of the following three reasons: ‘Financial reasons’, ‘Waiting list’ and ‘Too far to travel’ [22]. The European Observatory on Health Systems and Policies are using unmet medical need and forgone health care as synonyms [23] [24]. Both terminologies are used commonly in the literature. In our paper we are referring to unmet need and forgone care as synonyms.
